# Naming in a multilingual context: Norms for the ICMR-Manipal colour picture corpus in Kannada from the Indian context

**DOI:** 10.3758/s13428-024-02439-8

**Published:** 2024-06-24

**Authors:** Rajath Shenoy, Lyndsey Nickels, Gopee Krishnan

**Affiliations:** 1 Department of Speech and Hearing, Manipal College of Health Professions, Manipal Academy of Higher Education, Manipal, India; 2 School of Psychological Sciences, Macquarie University, Sydney, Australia

**Keywords:** Picture corpus, Bilingual, Multilingual, Kannada

## Abstract

There have been many published picture corpora. However, more than half of the world’s population speaks more than one language and, as language and culture are intertwined, some of the items from a picture corpus designed for a given language in a particular culture may not fit another culture (with the same or different language). There is also an awareness that language research can gain from the study of bi-/multilingual individuals who are immersed in multilingual contexts that foster inter-language interactions. Consequently, we developed a relatively large corpus of pictures (663 nouns, 96 verbs) and collected normative data from multilingual speakers of Kannada (a southern Indian language) on two picture-related measures (name agreement, image agreement) and three word-related measures (familiarity, subjective frequency, age of acquisition), and report objective visual complexity and syllable count of the words. Naming labels were classified into words from the target language (i.e., Kannada), cognates (borrowed from/shared with another language), translation equivalents, and elaborations. The picture corpus had > 85% mean concept agreement with multiple acceptable names (1–7 naming labels) for each concept. The mean percentage name agreement for the modal name was > 70%, with *H*-statistics of 0.89 for nouns and 0.52 for verbs. We also analyse the variability of responses highlighting the influence of bi-/multilingualism on (picture) naming. The picture corpus is freely accessible to researchers and clinicians. It may be used for future standardization with other languages of similar cultural contexts, and relevant items can be used in languages from different cultures, following suitable standardization.

## Introduction

Pictures are a valuable tool in cognitive science and kindred disciplines. They are employed both in experimental research to understand the nuances of various cognitive processes (e.g., language, attention, memory; for review, see Souza et al., 2020) and in various clinical populations (for instance, in dementia: e.g., Cuetos et al., 2005, 2012; Moayedfar et al., 2021; Silagi et al., 2015; and in aphasia: e.g., Alyahya et al., 2020; Bemani et al., 2022; Bose & Schafer, 2017; Brysbaert & Ellis, 2016; Cuetos et al., 2002; Nickels, 1995; Nickels & Howard, 1995). For all such purposes, pictures need to be carefully developed and evaluated in terms of various (psycholinguistic) attributes that are known to influence their processing across tasks.

In the past, several validated corpora of pictures have been made available in many languages and for specific purposes/populations (e.g., Bonin et al., 2003; Brodeur et al., 2010, 2014; Dunabeitia et al., 2018; Hebart et al., 2019; Moreno-Martínez & Montoro, 2012; Szekely et al., 2004; Szymanska et al., 2019). A closer inspection of these corpora shows that they are of two types: specific-purpose and general-purpose. Specific-purpose corpora are developed and validated for specific populations or purposes. For instance, the Besancon Affective Picture Set for Adults (BAPS-Adult) is a corpus of 256 attachment-related pictures (Szymanska et al., 2019), and the Image Stimuli for Emotional Elicitation (ISEE: Kim et al., 2018) is a set of 158 images that should elicit emotionality. Similarly, the Australian Beverage Picture set (Onie et al., 2020) was specifically developed to investigate cognitive bias in the Australian alcoholic population and therefore contains pictures of beverages (9 alcoholic and 10 non-alcoholic) across seven contexts.

General-purpose corpora, on the other hand, usually contain a large set of pictures that have been rated on various psycholinguistic properties such as imageability, familiarity, and visual complexity, and evaluated for name agreement with a group of speakers of the language under inquiry. The pictures in such corpora often include object pictures to elicit nouns that belong to common categories such as vehicles, vegetables, animals, and clothing (e.g., Bonin et al., 2020; Brodeur et al., 2010, 2014; Dunabeitia et al., 2018; Hebart et al., 2019; Snodgrass & Vanderwart, 1980; Szekely et al., 2004), and, less commonly, action pictures to elicit verbs (e.g., Ahmed et al., 2022; Szekely et al., 2004). The standardized set of 260 object pictures developed and validated by Snodgrass and Vanderwart (1980) is one of the most widely used general-purpose corpora for English. The original black and white line drawings have now been supplemented by coloured versions of these pictures (e.g., Rossion & Pourtois, 2004) and many more recent corpora have used coloured photographs (Brodeur et al., 2010, 2014; Hebart et al., 2019). Colour in particular has been found to improve picture naming accuracy, particularly for items with diagnostic colours and similar shapes, such as fruit and vegetables (Rossion & Pourtois, 2004; Bramão et al., 2011).

Pictures are visual instantiations of objects, actions, or entities in the world and are thus non-linguistic. Nevertheless, there are a few caveats in using a universal set of pictures around the world and across languages. First, objects depicted in a corpus may not be equally familiar to speakers of different languages, or to speakers of the same language across geographical areas. For instance, concept familiarity ratings for “baseball bat” showed that this item is more familiar among English speakers in the United States of America (Snodgrass & Vanderwart, 1980) than Spanish speakers in Spain (Sanfeliu & Fernandez, 1996). Second, the name agreement of objects can vary considerably across languages. For instance, for the same set of pictures, Spanish speakers elicited more different unique names per object (thus lower name agreement and higher *H*-statistic: Sanfeliu & Fernandez, 1996) than English speakers (higher name agreement and lower *H*-statistic: Snodgrass & Vanderwart, 1980). It is thus obvious that both item-level (e.g., item familiarity) and language-level (e.g., name agreement) factors can influence the attributes of a corpus. Such between–language differences have fuelled several adaptations of popular picture corpora. For example, adapted (or extended) versions of the Snodgrass and Vanderwart corpus have been published in Spanish (Sanfeliu & Fernandez, 1996), French (Alario & Ferrand, 1999), Icelandic (Pind et al., 2000), Portuguese (Pompéia et al., 2001), Japanese (Nishimoto et al., 2005), Malayalam (George & Mathuranath, 2007), Turkish (Raman et al., 2014), Argentinian Spanish (Manoiloff et al., 2010), Tunisian-Arabic (Boukadi et al., 2016), and Kannada (Bangalore et al., 2022). Similarly, Duñabeitia and colleagues (Duñabeitia et al., 2022) published a corpus of 500 colour pictures with data for name agreement and familiarity in 32 languages. While such corpora are of enormous importance for helping to understand the differences in language usage across the speakers of various languages, they nevertheless do not take into account the fact that many languages can reside in one speaker. That is, the speakers’ knowledge of more than one language and the interaction among those languages (while engaged in a psycholinguistic task) has not received sufficient research attention. Currently, an abundance of evidence from the bilingualism literature posits that all the languages of a speaker are active while engaged in a linguistic task (e.g., Brysbaert, 1998; Moon & Jiang, 2012; Van Hell & Dijkstra, 2002). In this context, we believe that gathering data in a truly multilingual context is important to inform us not only about the between–language differences among the speakers of various languages but also within speakers of more than one language (i.e., bi-/multilinguals). The current study is an attempt to explore such influence from an arguably ideal multilingual context—India.

India is home to 121 major languages (Census of India, 2011). In most geographical states of India, several languages are commonly spoken in addition to the primary language of that state. For instance, Kannada is the primary language spoken in the southern Indian state of Karnataka. However, other languages that are spoken in various parts of Karnataka include Tulu and Konkani (in the south-western sectors), Marathi (in the north-western parts), and Telugu and Tamil along the borders of Telangana and Tamil Nadu states, respectively (Census of India, 2011). Moreover, many individuals also speak Hindi and/or English (both official languages, and from the latter other languages have many lexical borrowings). Such linguistic diversity is expected to influence the ratings of psycholinguistic attributes and, more importantly, provide an opportunity to understand the influence of multilingual contexts on norms, something that is seldom considered in earlier studies. Hence, in this study, we developed a corpus of pictures and collected normative data with (multilingual) Kannada speakers in Karnataka.

There are already some published picture corpora in Kannada: United Nations Children's Fund (UNICEF) picture cards (simple line drawings: 361 semantic items, 303 syntactic structures; Karanth, 2007); a subset of the coloured version of the Snodgrass and Vanderwart (1980) picture set (*n* = 180, Bangalore et al., 2022); and coloured verb pictures (*n* = 269 based on their argument structure, Ahmed et al., 2022). However, only one of these sets (the UNICEF pictures, Karanth, 2007) are original and culturally curated coloured pictures suitable for Kannada speakers (and speakers of other Indian languages), and this set does not have name agreement or rated psycholinguistic attributes.

Duñabeitia et al. (2022) argue that, in general, to avoid restricting experimental design picture corpora need to be considerably larger than 300 items. Further, several researchers (e.g., Bangalore et al., 2022; Duñabeitia et al., 2022) have acknowledged the limitations of using black and white images, as they can affect the ease of recognition of the pictures (and thus the concepts). Indeed, only one of the available sets in Kannada (i.e., Bangalore et al., 2022, using Rossion & Pourtois, 2004 colour pictures) comprises coloured pictures, and it contains only 180 culturally appropriate pictures. Consequently, we aimed to develop an extensive, open-source picture database suitable for cognitive, linguistic, and clinical purposes with ratings on major psycholinguistic attributes in Kannada. We also aimed to explore the nature of naming labels when the normative data are collected from a multilingual population. To capture language-level variability, we use two name agreement measures: percentage name agreement and *H*-statistics. While percentage name agreement provides an index of agreement on a single name (modal name), the *H*-statistic captures variability in responses which arises from the interparticipant variability (Snodgrass and Vanderwart, 1980). For instance, two pictures could both have the most common name produced 70% of the time; however, one picture only has one alternative name produced, while another has five alternative names. The first picture has less variability in its naming responses, and this is reflected in a low *H*-statistic (*H* = 0.88). The second picture has more variability in naming responses (despite an identical number of participants [70%] producing its modal name), and a higher *H*-statistic (*H* = 1.47).

## Method

The study was approved by the Institutional Ethics Committee, Kasturba Medical College, Manipal Academy of Higher Education, Manipal (IEC: 178/2020). All participants recruited to this study read the participant information sheet and provided written informed consent before participating.

### Stimulus selection

As the motivation behind the development of this corpus was for a large bank of stimuli to be appropriate for use with adults with aphasia, the selection of concepts was considered with care, to ensure the clinical and functional relevance of the stimuli (e.g., Renvall et al., 2013a, 2013b). Consequently, we based the list of items on Palmer et al. (2017) who examined the word lists chosen for therapy by a large number (*N* = 100) of English-speaking people with aphasia from the UK and provided lists of the most common topics and words. However, to make the corpus suitable for usage in the Indian multilingual and multicultural context, while some items that were relevant across cultures (*n* = 605 nouns) were selected from the Palmer et al. list (e.g., pen, chair), others were more relevant to a UK population (such as specific buildings like Blenheim Palace). These items were replaced with culturally specific items (*n* = 64; e.g., monuments: Indian Parliament, Taj Mahal, Mysore Palace; food items: *idly*, *upma*). Of the 669 object pictures, 508 depicted human-made objects and 161 represented natural objects^1^. In addition to the 669 nouns, 99 picturable verb names^2^ were selected from an existing Kannada word repository (Prarthana, 2015). We chose to develop coloured images rather than black and white, because of the facilitating effect of colour on naming accuracy (Rossion & Pourtois, 2004).

A hired digital artist developed the pictures corresponding to the nouns and verbs using a professional software program (Adobe Photoshop CS3). We instructed the artist to draw images in a way that was appropriate for both young and older adults as well as a multicultural audience. All pictures were drawn on a white background. However, a few (e.g., coast, star, lightning, moon) needed background colours to enrich the target image and to reduce ambiguity. All pictures had a uniform dimension (height × width: 2480 × 3508 pixels) and were saved in .jpg format (see sample pictures in Appendix Figs. 3 and 4). Before they were used for subsequent ratings, the authors inspected them for quality and clarity and requested a revised version from the artist for those pictures that were deemed inappropriate.

### Norms

#### Participants

We recruited 120 native adult speakers of Kannada from the Udupi district of Karnataka through a word-of-mouth (snowballing) approach. Participants self-reported that they were fluent speakers of Kannada. Additionally, all participants were multilingual, reporting that they were fluent in a minimum of two languages (mean = 3.6: *SD* = 0.81, median = 4: IQR = 1, range = 2–5) (see Supplementary Material S1 for the language status of the participants). Participants had a minimum of 7 years of education and were able to read and write Kannada^3^. None had any current (or history of) cognitive, language, or neurological disorders. The participants were divided into four groups (30 per group, following Snodgrass & Vanderwart, 1980) to carry out the following tasks: (1) name agreement for noun pictures (mean age = 22.4: *SD* = 2.7 years); (2) name agreement for verb pictures, (mean age = 20.5: *SD* = 0.6 years; (3) image agreement ratings for both nouns and verbs (mean age = 20.3: *SD* = 0.7 years); and (4) ratings of familiarity, (subjective) frequency, and age of acquisition (mean age = 39.3; *SD* = 13.3 years).

### Procedure

#### General

This study was carried out in a hybrid mode (i.e., online and offline, due to COVID-related restraints). The name and image agreement ratings were conducted online. The rating of psycholinguistic variables was conducted in offline mode (except for five participants who did the rating online). For all the online participants, an orientation session was carried out over video conference to introduce the purpose of the study, and to highlight the importance of adhering to the instructions. The pictures were presented by screen-sharing a Microsoft PowerPoint presentation to all online participants simultaneously. Adequate time was provided to respond to each item in each task (e.g., ~1 minute to respond in the name agreement task, ~30 seconds to respond in the image agreement task). Participants were asked to write their responses on paper and later send a scanned copy to the first author. The entire online rating process lasted for five 2-hour sessions for each task (i.e., name and image agreement). Those participants who missed any online session received additional session(s) to complete the rating of missed items. All participants were asked to note their participant identifier, age, gender, languages known and spoken, and educational background on the response sheet.

#### Name agreement

As mentioned above, the name agreement task was carried out online. Each picture was displayed individually on a Microsoft PowerPoint slide. Participants were instructed to look at the picture and write down the first name (in Kannada) that came to mind on a sheet of paper (along with the slide number). They were allowed to respond with single, compound, or multiple words. Participants who named noun pictures of objects were required to use a noun, and those who named action pictures were instructed to use a verb in their response. Further, the participants were instructed to write “DKO” (i.e., Do not Know the Object/Verb) if they did not know the object/action, “DKN” (i.e., Don’t Know the Name) if they knew the object/action but not the name, and “TOT” (i.e., Tip-of-the Tongue) if they believed they knew the object/action and its name but were unable to retrieve the name momentarily. Considering the transparency of the Kannada script (highly consistent syllable–grapheme correspondence; Karanth, 2003), the participants were requested to write the picture names in the Kannada script (even for borrowed words from other languages). After completing the name agreement task, the participants were requested to scan and send their responses to the first investigator via e-mail or shareable drive.

### Image agreement

The modal names (i.e., the most commonly produced correct names) of the pictures from the name agreement data were used for image agreement ratings. The investigator displayed the modal name (using Microsoft PowerPoint) while reading it aloud. Participants were instructed to listen to the spoken name (by the investigator) and read the displayed name, and create a mental image of the concept. After a gap of 5 seconds, the stimulus picture was displayed. Participants were required to rate the agreement between their mental image and the displayed image on a five-point Likert scale (1 – no match, 2 – poor match, 3 – average match, 4 – high match, and 5 – perfect match).

#### Ratings for other pertinent psycholinguistic variables

Ratings for other relevant psycholinguistic variables (see below) for the nouns and verbs were collected for correct responses (see below for the definition of correct response; total *n* = 1650 nouns for 669 object pictures, 146 verbs for 99 action pictures) produced in the name agreement rating. The participants were briefed by the first author about the study and the task requirements. They were provided with the stimulus and response booklets (or the link to an online response using Google Forms) and given 15–20 days to complete the tasks. Five participants completed the rating using Google Forms and the rest completed the task offline, returning the booklet to the investigator. Reminder messages to complete the ratings were sent periodically (every ~5 days) to participants’ mobile phones. Incomplete entries were identified and returned to the participants to complete and resend.

The participants were required to rate each stimulus on the following psycholinguistic variables:

1. Word familiarity: The participants rated each word using a five-point Likert scale (1: unfamiliar, 2: less familiar, 3: moderately familiar, 4: familiar, 5: very familiar) on how commonly they heard that word in their spoken language (Brown & Watson, 1987; Gilhooly & Logie, 1980).
2. Subjective frequency: As there are no subjective frequency counts for Kannada words, following Desrochers and Thompson (2009), we asked the participants to rate each word on its frequency of usage in daily communication using a five-point Likert scale (1: never; 2: rarely; 3: occasionally; 4: frequently; 5: very frequently).
3. Age of acquisition (AoA): For this task, the participants were instructed to rate the approximate age at which each word was acquired. Following Gilhooly and Logie (1980), we used a seven-point Likert scale (1: < 2 years; 2: 2–3 years; 3: 3–4 years; 4: 4–5 years; 5: 5–7 years; 6: 7–10 years; and 7: > 10 years).

Additionally, we calculated the number of syllables for all correct names. Note that for loan words, we have provided the syllable counts of both the loan words and their lexicalized forms in Kannada (e.g., syllable counts for pen in English (/peɳ/) is 1, and in Kannada (/pennu/) is 2). Supplementary files S2.xlsx for nouns and S3.xlsx for verbs provide the descriptive statistics for the psycholinguistic variables for every correct response in the corpus.

We also report the objective visual complexity of the images, obtained by retrieving the file size (in KB) of each of the images (see Mirman et al., 2010; Székely & Bates, 2000, for similar measures).

### Analyses

Following the collection of name agreement data, we cleaned the data by correcting the typos in the written responses. For nouns, subsequent preprocessing included collating basic variants of the same names such as plurals (e.g., pan, pans) and orthographic variations (e.g., {  (pennu) /  (pen) International Phonetic Alphabet [IPA]: pen̪n̪u /pen̪/}). For verbs, we considered only the root form (e.g.,  (helu) IPA: /ɦeːɭu/ meaning “tell”) and discarded all other agglutinative morphosyntactic variations (e.g.,  (helutidaane; IPA: /helutid:ne/,  (helutidaale; IPA: /helutida:Le/,  (heluvudu; IPA: ɦeːɭuʋud̪u/ were all marked  (heLu) IPA /ɦeːɭu/).

#### Concept agreement

We first determined whether the concept referred to by the naming responses corresponded to the target depicted. For example, in English, “sofa”, “settee” and “couch” are all correct possible labels for the same concept. In contrast, the label “plastic glass” cannot be used as an adequate label for the concept of “garbage bin” (O’Sullivan et al., 2012). Following previous similar studies (e.g., Duñabeitia et al., 2018; O’Sullivan et al., 2012), when the depicted concepts corresponded with their names, we categorized them as correct (i.e., acceptable labels for the concept) and, when they did not, they were grouped as incorrect (i.e., labels that did not correspond to the concept displayed in the picture). Incorrect responses included semantically related (yet distinct entities: e.g., “harmonium” for “accordion”), and visually similar (yet semantically distinct entities: e.g., “banana” for [crescent] “moon”). The (rare) occasions of uncertainty regarding whether or not a label corresponded to a concept were resolved with the help of two native Kannada speakers considering the colloquial usage of such words (especially for loan words from English).

After eliminating the incorrect responses, the correct name(s) of each concept were rank-ordered. The most common (correct) name provided for a concept was taken as the modal name. Supplementary S2.xlsx and S3.xlsx file provides the raw frequency and percentage of the name(s) produced for each concept.

In contrast to the previous literature that provided name agreement data of the modal names alone, we provide all the correct names produced for each concept and their rate of occurrence. Further, we provide the ratings for word familiarity, subjective frequency, and age of acquisition for every correct name of the concepts.

The first author and three native Kannada speakers (all four being multilingual), classified the correct names into the following categories:

i. Target language names: Kannada words (e.g., “  ” (kannu) IPA /kəɳɳu/ “eye”).
ii. Cognates: Words that phonologically overlap with a word in another language (e.g., English) and where there was no unique name for that concept^4^ in the target language. These words could be loan/borrowed words that were a) modified (e.g., “  ” /pen̪n̪u/ for “pen”), b) one of the components of a compound (e.g., “  ” (/ka:ru nilugade/) for car parking), or c) not modified (e.g., “  ” for laptop).
iii. Translation–equivalents: This category was similar to cognates except for concepts where there was also a target-language name. For instance, the Kannada name for the concept of “television” (which is often used in a shortened form as ‘T.V.’) is “  ” (du:rdarshana)^5^. Similarly, the verb “kill” is (“  ” (kolluvudu) in Kannada, but the word “  ” “murderma:du” derived from “murder” in English, was among the labels produced.
iv. Elaboration: This category a included number of different response types all of which were relatively infrequent in the noun data and did not occur in the verb data.  Examples include i)  literal translations into Kannada of a (compound) name in another language (e.g., “bathtub” produced as  “  ” (/sna:nada/ - bath) “  ” (/totti/ - tub), where that lexical item does not exist in Kannada (the Kannada word would be the cognate “bathtub” “  ”; or ii) responding with a category name when a unique word in the target language was unavailable. For example, there is no single lexical item for “saxophone” in Kannada and this was often named using the Kannada word for musical instrument (  - /va:dya/). A possible influence of the instructions to name the entities in Kannada might have yielded such responses. Though such responses were rare in the name agreement data, they were considered correct for concept agreement.

Commonly reported measures of name agreement data include the modal name and the *H*-statistic (an index of homogeneity of names produced: Snodgrass and Vanderwart, 1980). We report these measures calculated for nouns and verbs. We calculated the *H*-statistic using the following formula ***H*** = ∑***i*** = **1*kpi*** log**2**(**1**/**pi**), where k refers to the number of different responses to a picture across all participants, and p refers to the proportion of responses given for each name of a concept (Snodgrass & Vanderwart, 1980). The lowest value of the *H*-statistic is 0 (i.e., perfect name agreement: only a single name for a given concept), and a higher *H* value indicates the multiple names for that concept (Snodgrass & Vanderwart, 1980). Snodgrass and Vanderwart (1980) calculated the *H*-statistic by including all naming responses, excluding “IDK” and “TOT” responses. There is a lack of clarity in the literature on whether to include incorrect labels referring to a familiar concept (e.g., banana for crescent moon) or an unfamiliar concept (e.g., harmonium for accordion) in the calculation of *H*-statistics. However, George and Mathuranath (2007) excluded incorrect naming labels during *H*-statistic calculation. Hence, we calculated the *H*-statistic in two ways: (i) including all the names (i.e., correct and incorrect names, but excluding IDK and TOT responses) and (ii) including only the correct names.

Similarly, we calculated two measures of percentage agreement: (i) percentage picture–concept agreement—the percentage of responses that corresponded to the concept depicted by the picture (excluding IDK and TOT responses, similar to Snodgrass & Vanderwart, 1980; Singh et al., 2020) and (ii) percentage name agreement for all correct names. In line with other studies providing normative ratings (Bangalore et al., 2022; Brodeur et al., 2014; Duñabeitia et al., 2018; George & Mathuranath, 2007; Singh et al., 2020; Snodgrass & Vanderwart, 1980), we also examined the correlations between the psycholinguistic variables. The results of these analyses are provided in the Appendix (see Table 5 for nouns and Table 6 for verbs).

Additionally, we examined the variability of ratings across participants for the subjective ratings (image agreement, familiarity, subjective frequency, age of acquisition). To do this, we analysed the modal names of nouns (*n* = 663) and verbs (*n* = 93) using two methods. The first method used the intraclass correlation coefficient (ICC; Shrout & Fleiss, 1979, also see similar methods in Momenian et al., 2021) to examine the reliability of participants’ ratings (*n* = 30). We considered a two-way mixed model with an average rating selection (see Koo & Li, 2016; Shrout & Fleiss, 1979). The second method used split-half correlations in which we compared the ratings of the 30 participants divided into two groups (Group 1: even-numbered participants and Group 2: odd-numbered participants; for a similar method see Decuyper et al., 2021).

## Results

The summary statistics for the nouns and verbs are provided in Tables 1 and 2, respectively. Supplementary S2.xlsx provides each noun picture’s (1) image agreement data, (2) *H*-statistic for all names (correct and incorrect), (3) *H*-statistic using only correct names, (4) concept agreement data, and (5) correct names in the descending order of occurrence with their frequency and the IPA transcription of each name. Supplementary file S3.xlsx provides the same data for the individual verb pictures.

**Table 1:** Summary statistic for nouns (*n* = 663)

							Skewness		Percentiles	
	Mean	Median	*SD*	IQR	Minimum	Maximum	Skewness	*SE*	25th	75th
Concept agreement (%)	90.64	100.00	20.55	6.67	3.33	100.00	−2.59	0.09	93.33	100.00
Objective visual complexity (KB)	1059.28	986.46	392.83	357.03	26.39	3845.39	1.90	0.09	848.55	1205.59
Modal name (%)	71.24	76.67	25.19	40.00	3.33	100.00	−0.64	0.09	53.33	93.33
*H*-statistic: all labels	0.89	0.90	0.66	1.02	0.00	2.92	0.31	0.09	0.35	1.38
*H*-statistic: correct concept labels	0.72	0.67	0.60	1.20	0.00	2.20	0.34	0.09	0.00	1.20
Image agreement	4.00	4.00	0.16	0.20	2.63	4.50	−0.91	0.09	3.90	4.10
Familiarity	3.11	3.10	0.40	0.40	1.40	4.83	0.60	0.09	2.90	3.30
Frequency	2.98	3.00	1.08	1.87	1.00	4.73	−0.16	0.09	2.10	3.97
Age of acquisition	4.85	5.03	1.31	2.13	1.33	6.70	−0.48	0.09	3.87	6.00
Syllable count	2.62	2	1.27	1.00	1	10	1.33	0.09	2.00	3.00

^1^Image agreement, familiarity and frequency were rated on a five-point rating scale. Age of acquisition was rated on a seven-point rating scale

^2^Concept agreement (%) and modal name (%) are calculated as a percentage of responses

^3^Objective visual complexity is based on file size in KB

**Table 2:** Summary statistic for verbs (*n* = 96)

							Skewness		Percentiles	
	Mean	Median	*SD*	IQR	Minimum	Maximum	Skewness	*SE*	25th	75th
Concept agreement (%)	86.42	100.00	24.37	17.50	13.33	100.00	−1.84	0.24	82.50	100.00
Objective visual complexity (KB)	1130.58	1129.29	244.99	271.63	414.77	1722.36	−0.25	0.24	990.26	1261.90
Modal name (%)	78.05	91.66	26.48	40.00	6.67	100.00	−1.01	0.24	60.00	100.00
*H*-statistic: all labels	0.52	0.28	0.56	0.98	0.00	1.87	0.52	0.24	0.00	0.98
*H*-statistic: correct concept labels	0.28	0.00	0.47	0.56	0.00	1.83	1.39	0.24	0.00	0.56
Image agreement	3.59	3.53	0.95	1.01	1.40	4.57	0.28	0.24	3.07	4.08
Familiarity	4.50	4.50	0.10	0.11	4.27	4.73	0.21	0.24	4.43	4.54
Frequency	4.36	4.47	0.39	0.17	2.43	4.70	−2.88	0.24	4.40	4.57
Age of acquisition	3.93	3.55	0.88	0.67	2.87	6.47	1.50	0.24	3.39	4.07
Syllable count	2.52	2.00	0.99	1.00	1.00	5.00	0.81	0.24	2.00	3.00

^1^Image agreement, familiarity and frequency were rated on five-point rating scale. Age of acquisition was rated on a seven-point rating scale

^2^Concept agreement (%) and modal name (%) are calculated as a percentage of responses

^3^Objective visual complexity is based on file size in KB

### Nouns

#### Concept agreement

We removed six noun pictures from the 669-item corpus due to poor concept agreement (i.e., burglar alarm, chemist, Horlicks, rug, sailing, tumble dryer), as they did not elicit any names corresponding to the target concept. For the remaining 663 pictures, the mean concept agreement was 90.64% (SD: 21.3%). The incorrect names and IDK responses constituted 4.27%, and names referring to the incorrect concepts comprised 5.09% of responses in the noun corpus. The majority of the items (n = 561; 84.6%) had concept agreement greater than 80%. Of the remaining 102 pictures, 34 had concept agreement between 61–80%, 30 between 41–60%, 22 between 21–40%, and 16 pictures below 20%.

#### Name agreement

**Fig. 1:**
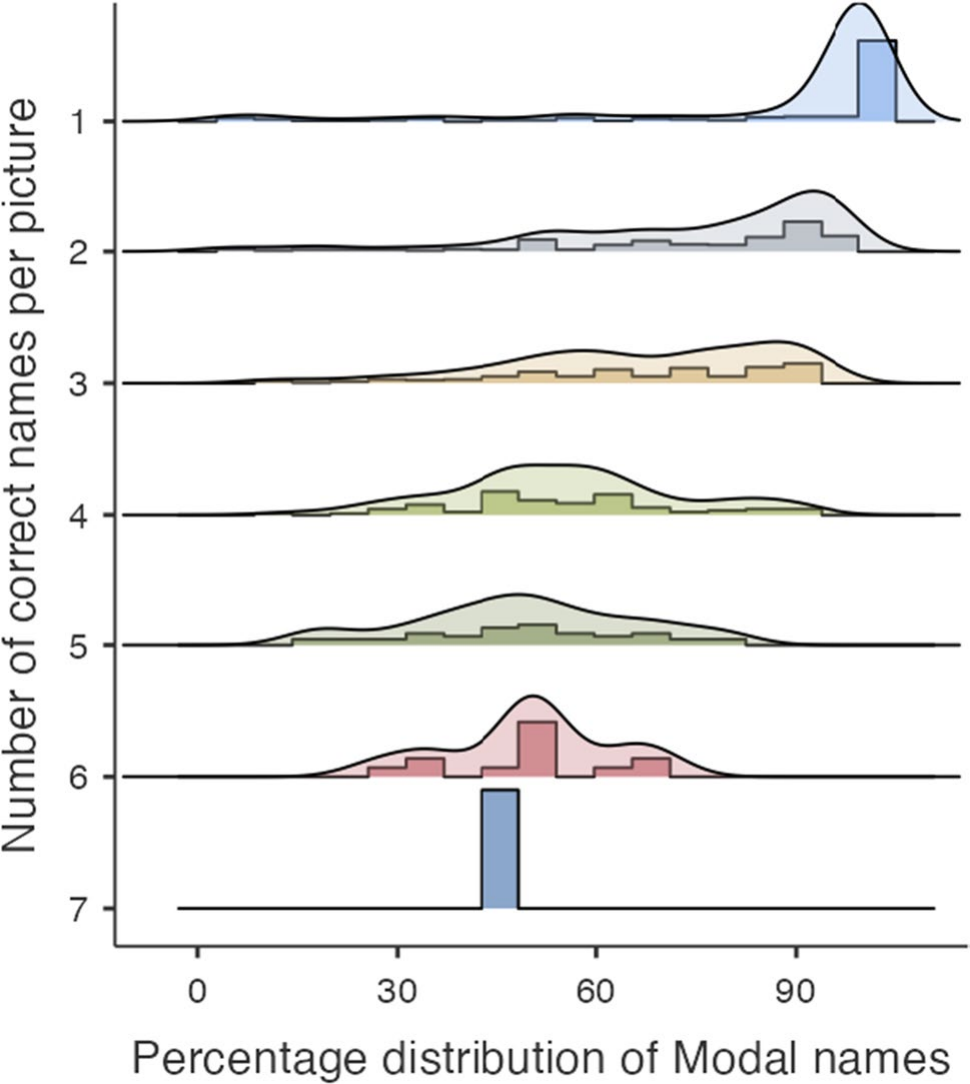
The percentage distribution of the modal names as a function of the number of correct names per picture. *Notes:* Of the 663 pictures, 172 had a single name, 199 had two names, 156 had three names, 81 had four names, 41 had five names, 13 had six names and one picture had seven names

The mean percentage of participants who used the modal (i.e., correct and most frequently produced) name for the concept was 71.24% (*SD* = 25.19%). Table 1 provides the summary statistics at the group level for the modal nouns. The *H*-statistic for the nouns in this corpus was 0.89 (SD: 0.66) for all (correct and incorrect) labels, and remained high at 0.72 (*SD* = 0.66) when only correct labels were included. There was high variability in correct naming responses, with up to seven names for a picture. Of the 663 nouns, 25.9 % (i.e., 172 pictures) had single names, 30.01 % (i.e., 199 pictures) had two names, and 23.52 % (i.e., 156 pictures) had three names. Figure 1 depicts the percentage distribution of the modal names for pictures with 1–7 names.

#### Image agreement

The mean image agreement rating of the noun corpus was 4.00 (*SD* = 0.16), with ratings for all but one noun (accordion: mean = 2.63, *SD* = 1.38) greater than 3. These relatively high mean image agreement ratings suggest that the noun pictures accurately reflected the stereotypical image of the modal names.

## Familiarity, frequency, age of acquisition ratings, and syllable counts

The mean ratings of the modal names for familiarity, frequency, and age of acquisition are provided in Table 1. Though all modal names were familiar (scale of 1–5: mean = 3.1; *SD* = 0.4), they had a relatively low mean subjective frequency (scale of 1–5: mean = 2.9; *SD* = 1.3) and a relatively high age of acquisition (scale of 1–7: mean = 4.85; *SD* = 1.3). The modal name for the nouns had a mean syllable count of 2.62 (*SD* = 1.27).

### Correlations between variables (nouns)

For nouns (see Appendix Table 5) the percentage of modal names (i.e., name agreement) showed a significant correlation with all other variables except for image agreement: items with higher name agreement and a lower *H*-statistic were more familiar, more frequent, lower in age of acquisition, had fewer syllables and were less visually complex. Concept agreement showed the same pattern, except that the correlation with the number of syllables was not significant.

In line with previous reports (Alario & Ferrand, 1999; Pompéia et al., 2001; Pind et al., 2000; Sanfeliu & Fernandez, 1996; Snodgrass & Vanderwart, 1980; Wolna et al., 2022), and as expected, we found negative correlations between the *H*-statistic and the percentage name agreement in the noun corpus. The nouns showed significant correlations between the psycholinguistic variables, except for image agreement which did not show any significant correlation with the remaining variables. This indicates that the extent to which the noun images reflected participants’ stereotypical view of that item was not influenced by any of the other factors. As would be expected, familiarity and frequency showed significant positive correlations with each other, and negative correlations with the AoA and the number of syllables. Similarly, as expected, the AoA and the number of syllables were positively correlated.

### Verbs

#### Concept agreement

From the initial set of 99 verb pictures, we removed three items (remember, spend, fry) that did not elicit the target concept. The mean concept agreement for the remaining 96 verbs was 86.4% (*SD* = 24.3: range: 13.3–100%). The mean percentage of “I don’t know” responses was 2.5% (*SD* = 6.5). As for nouns, the majority of verb pictures (75%; *n* = 72) had concept agreement greater than 80%. Of the remaining 24, nine verbs had concept agreement ratings of between 80 and 61%, seven ratings of 60–41%, three of 40–21%, and five verbs of less than 20%.

#### Name agreement

Compared with nouns, the verbs had slightly higher mean percentage name agreement (modal names: mean = 78.05%; *SD* = 26.48) and lower mean *H*-statistic (with all responses: *H* = 0.52 [*SD* = 0.56]; with correct responses: *H* = 0.28 [*SD* = 0.47]). The number of correct names produced for verb pictures ranged between 1 and 4. Sixty-five verb pictures (68%) had only single names. Table 2 provides the summary statistics of the modal verbs.

#### Image agreement ratings

The mean image agreement of the verb corpus was 3.59 (*SD* = 0.95). This relatively high rating suggests that the newly developed verb pictures largely depicted the stereotypical images of the modal names.

## Familiarity, frequency, age of acquisition ratings, and syllable counts

All modal verb responses were highly familiar (rated over 4 on a scale of 1–5) and were also of relatively high mean subjective frequency (4.63, scale of 1–5). The mean age of acquisition was 3.93 years (scale of 1–7). Thus, the modal verb names had higher familiarity and subjective frequency, and lower AoA compared with the modal nouns. The modal name for the root verbs had a mean syllable count of 2.52 (*SD* = 0.99).

### Correlations between variables (verbs)

For verbs (Table 6), percentage name agreement (modal name %) and the *H*-statistic were, as expected, negatively correlated with each other, but not significantly correlated with any other variable except for concept agreement. The only other significant correlations were between the age of acquisition and image agreement, with later-acquired verbs having higher image agreement, and a negative correlation between familiarity and number of syllables (verbs with fewer syllables were more familiar).

#### Reliability ratings

Appendix Table 8 provides the results of the ICC analysis (Shrout & Fleiss, 1979) using Koo and Li’s (2016) guidelines to interpret the correlation coefficients. Table 9 provides the results of the split-half correlation. For both nouns and verbs, the subjective frequency and age of acquisition ratings showed “excellent” ICC (> 0.90) and high split-half correlations (> .8). In terms of image agreement ratings, the verbs showed high ICC and split-half correlations (> 0.90), whereas the nouns showed poor reliability (ICC < 0.15; split-half < .05). Familiarity ratings for nouns showed moderate reliability (ICC > .6, split-half > .5) though the verbs revealed poor reliability (ICC < .2; split-half < .15).

## Variability in the naming labels

### Nouns

**Fig. 2:**
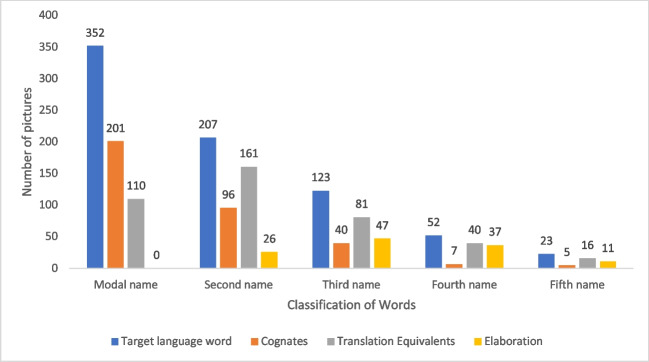
Number of pictures with their distribution of correct response types. *Note:* 352 pictures had the target language word as the modal name. Similarly, 201 pictures had cognates and 110 pictures had translation-equivalents as the modal names

Table 3 provides the frequency of occurrence of each response type (i.e., target language word, cognates, and translated equivalent) of modal names which were man-made and natural objects. Figure 2 provides word classifications (target language word, cognates, translated equivalent and elaboration) across acceptable alternatives (i.e., modal name, second name for the picture [CN2], third name of the picture [CN3]). We also provide some descriptive observations and examples in Table 4. It is of particular note that there were strong cross-linguistic influences, with 110/663 instances (pictures which were modal names) of an English translation equivalent being used even when a Kannada word existed for the target (see Fig. 2).

**Table 3:** Modal name and word classification counts across human-made and natural kinds of objects

Modal name	Object	Counts	% of Total	Cumulative %
Target language word	Human-made	209	31.5 %	31.5 %
	Natural	143	21.6 %	53.1 %
Cognates	Human-made	189	28.5 %	81.6 %
	Natural	12	1.8 %	83.4 %
Translation equivalents	Human-made	104	15.7 %	99.1 %
	Natural	6	0.9 %	100.0 %

*Note:* There was no elaboration in the modal name

**Table 4:** Summary of variations and examples observed in the noun pictures

**Response characteristic**	**Examples and brief summary**
**The modal name is in the target language**	For example, for the concept “chair” (Kannada IPA /kuɾt͡ʃɪ/), every participant produced the same name in the target language
**The modal name and its synonyms are in the target language**	Example: For the concept “guava”, we received two naming labels. The modal name was “perale” IPA /peːɾəɭe/ (83.33%) and synonym “sebehannu” IPA: /s̪iːbeɦəɳɳu/ (16.67%). Both words are acceptable synonyms in the target language
**The modal name shares naming labels in the target language**	Balatsou et al., (2022) provided an example of “couch” and “sofa” as shared modal labels. In our corpus, the concept “knife” had two alternative labels, “chaku” IPA /t͡ʃɑːku/ and “churi” IPA /t͡ʃuːɾɪ/, with 50% name agreement for each
**The modal name is a cognate (no corresponding target language label)**	Example 1: For the concept “bread”, the response was IPA /{bɾeɖ / bɾeɖd̪u }/, the percentage of name agreement was 100%. Example 2: For the concept “screwdriver”, the response was IPA /s̪kɾuːɖɾəɪʋəɾ/ and had 100% name agreement
**The modal name and synonyms are cognates**	Example 1: For the concept “piano”, we received two naming labels. The modal name was a cognate IPA /pɪjɑːn̪o/ (name agreement of 86.6%) and the synonym was “keyboard” IPA: /kiːboːɾɖə/ (10%)
**The modal name is a “translation equivalent” word (target language word exists).**	For the concept carrot, the translated equivalent word /carrot/ IPA /kjɑːɾeʈ/ had a higher percentage of name agreement (86%) compared with the target language word “gajjari” IPA /gəd͡ʒd͡ʒəɾɪ/ (10%).
**Both “target language word” and “translation equivalents” are common labels.**	Example 1: For the concept “watch”, the target language word kai gadiyara IPA /kəɪ gəɖɪjɑːɾə/ had name agreement of 56% and the translated equivalent word “watch” or “watchu” IPA /ʋɑːt͡ʃ/ʋɑːt͡ʃu/ had name agreement of 44%. Example 4: For the concept “camel”, both the target language word “onte” IPA /õʈe/ and the translated equivalent word “camel” IPA /kjɑːməl/ had a percentage of name agreement of 50%.

### Verbs

Unlike in nouns, cross-lingual influences (e.g., from English) were minimal in verb names. We found only 7/96 instances of the use of translation equivalents in verb names (1: to get ready – “*readi*agudu” (  ) [IPA: ɾeːɖɪjɑːgud̪u]; 2: to dance – “*dance*madu” (  ) [IPA: ɖjɑːn̪s̪ mɑːɖuʋud̪u ]; 3: to kill – “*murder*m:adu” (  ) [IPA: məɾɖə mɑːɖuʋud̪u]; 4: to walk – “*walk*madutirodu” (  ) [IPA: ʋɑːkʰ-mɑːɖut̪t̪ɪɾoːd̪u ]; 5: to taste – “*taste*no:du” (  ) [IPA: ʈeːs̪ʈ n̪oːɖuʋud̪u]; 6: to fry – “*fry*ma:du” (  ) [IPA: pʰɾəɪ mɑːɖu]; 7: “*cut*ma:du” (  ) [IPA: kəʈ-mɑːɖud̪u] for the verb “cut”).

## Discussion

We have reported on the development of a set of 759 colour pictures (663 noun pictures and 96 verb pictures) that we believe will prove a valuable asset for use in both research and clinical domains. To make the corpus of maximal clinical utility, we based the domains from which target names were drawn on objective data from a study of functionally relevant items (Palmer et al., 2017). Subsequently, we included as many picturable nouns and verbs as possible that could be useful across different clinical populations and in experimental work with unimpaired populations.

This corpus helps rectify the issue that, in under-resourced languages, a practical challenge faced by many clinicians and researchers is the lack of availability of culturally curated picture corpora. The use of pictures from other cultures is often inappropriate and unacceptable (Ahmed et al., 2022). However, the development of a large picture corpus requires extensive resources including time, professional (including artistic) skills, and budget, and hence is often not feasible. The corpus, the ICMR-Manipal Colour Picture Corpus, provides a culturally curated resource of pictures with norms on name agreement, image agreement and psycholinguistic ratings that can be used directly among speakers of Kannada, a language spoken by nearly 50 million people (Census of India, 2011). We believe that the pictures from this corpus may also be appropriate and useful for clinical and research purposes, and, after validation, in other similar sociocultural–linguistic backgrounds.

Beyond the corpus as a resource, we highlight the importance of not only extending corpora to different languages but also examining them in multilingual contexts. The findings from the current study provide several insights into the influence of the multilingual context of the participants on both naming and psycholinguistic variables. Though several well-known picture corpora have been adapted to many languages (e.g., Snodgrass & Vanderwart, 1980), the data from such adapted corpora may fail to reflect the cross-linguistic interactions among those languages as participants rated the pictures from multiple *monolingual* contexts. Below, we discuss these aspects of the current study.

### Variability in naming

Substantial variability was found in the names produced by the multilingual speakers recruited here, particularly for nouns. This is reflected in the large number of correct names and the resulting high *H*-statistic (the *H*-statistic is an index of naming ambiguity—a picture with several names has a higher *H*-statistic: Snodgrass & Vanderwart, 1980).

#### Nouns

The majority of the noun concepts in the corpus had more than one correct name. The mean *H*-statistic for nouns (all names: 0.89; correct concept names: 0.72) was higher in our corpus relative to previous investigations with monolingual speakers (0.47: Turkish, Raman et al., 2014; 0.36: French, Alario & Ferrand, 1999; 0.36: Polish, Wolna et al., 2022) and multilingual speakers (0.66: Kannada, Bangalore et al., 2022; 0.54: Malayalam, George & Mathuranath, 2007) except for Hindi speakers (0.90: Hindi, Singh et al., 2020). Correspondingly, the mean percentage of the modal names for the nouns (71%) was less than that in previous similar studies with monolingual speakers (83%: Turkish, Raman et al., 2014; 85%: French, Alario & Ferrand, 1999; 91%: Polish, Wolna et al., 2022; 81%: Hindi, Singh et al., 2020). Together, the lower mean percentage values of the modal names and the higher *H*-statistic indicate higher variability in the naming responses. Brodeur et al. (2010) report that their colour picture corpus received a lower percentage of modal names (64%) and a higher *H*-statistic (1.65) than the line drawing corpora. They suggest that this could be due to the influence of colour details leading to more alternate names (e.g., “pepper” versus “red pepper”, see Brodeur et al., 2010, p. 7). The higher number of alternate names (see Fig. 1) and higher H statistics in the current corpus may be attributed to the usage of colour pictures like in Brodeur et al. (2010).

When examining the noun data, we noticed that the nature of the object appeared to influence the category of response. Indeed, when we divided the noun stimuli into those representing man-made concepts and those representing natural kinds, there was a clear difference (see Table 3) and there was a significant association between modal name classification and object categorisation (χ^2^ = 109, *df* = 2, *p* < 0.01). That is, the concepts of human-made objects were predominantly tagged with cross-lingual names, and labels of natural objects were predominantly in the target language. This could plausibly be attributed to the fact that the names of new technology-based devices (e.g., computers) were borrowed from English and later served as independent lexical entities in Kannada. However, for some of such borrowed words, unique target language words were available. For instance, it was evident that many of the household articles (e.g., furniture, utensils) were named with translations despite the availability of their unique names in Kannada. Speakers often seemed to prefer the borrowed words to the translated words. One possibility is that translation equivalents were preferred when they were shorter than the target language names. Indeed, 34% of the translation equivalent modal names were of one syllable, relative to only 4% of the Kannada modal names (see Appendix Table 7, χ^2^ = 83.514, *df* = 9, *p* < 0.01). In addition, the frequent use of translation equivalents suggests that, for multilingual speakers who are exposed to English, when the target language word for an object is momentarily unavailable, the knowledge of the English language may provide an alternative means to name the object.

#### Verbs

Compared with nouns, the verbs showed higher name agreement (verbs: 78%; nouns: 71%) and a lower *H*-statistic (all and correct responses of verbs: 0.52 and 0.28; of nouns: 0.89 and 0.72). Approximately a third of the verbs elicited only one name. In addition, the percentage of modal verb names was lower and the *H*-statistic was higher than those of another recent study in Kannada (Ahmed et al., 2022; mean *H*-statistic = 0.31 [*SD* = 0.47]; mean % of modal name = 90.32% [*SD* = 15.90], for three-argument verbs). However, the mean *H*-statistic for verbs in the current corpus was lower than that reported in a recent Polish study (Wolna et al., 2022; *H*-statistic: 0.89, mean % modal verb name: 79.39%).

We found that the cross-linguistic influence was less apparent in verbs compared with nouns. For instance, only 7/96 (7.3%) verbs showed translation equivalent names in contrast to 201/663 (30 %) nouns. While there is a rich body of literature on the differences between nouns and verbs at the conceptual level (e.g., Yang et al., 2017), these two grammatical classes seemed to experience different cross-linguistic influences. One possibility is that verbs are more difficult to replace as they undergo linguistic transformations. As the names of actions, verbs need additional morphemes to indicate the state of their occurrence. Especially in inflectional languages like Kannada, morphological and syntactic structures interact together to form complex morphosyntactic constructions. For example, the sentence “she is washing the vessels” in English would translate to “avaLu pa:tregaLu toLijuttida:Le”. Replacement of the same verb “wash” (“toLijuvudu”) with its English counterpart would require an *additional* morpheme (e.g., “avaLu pa:tragaLu clean *ma:duttida:Le*”). Thus, it might be the economy of expression (e.g., Dalrymple et al., 2016) that makes the verbs (action words) more resilient to cross-lingual interference than the nouns.

#### Cross-linguistic influences

As noted earlier, we believe that to better elucidate crosslinguistic influences, data should be collected from people who speak multiple languages rather than from monolingual speakers of multiple languages. In the current study, name agreement norms were collected from native Kannada-speaking university students whose medium of instruction was English. Engaging participants from a multilingual context to rate various attributes in a specific language provided us with the opportunity to capture the natural crosslinguistic interactions including, as discussed above, the use of translation equivalents despite the availability of labels in the target language. The increased variability in noun names in the current study relative to previous studies supports our argument. For instance, a given concept (e.g., table) in a multilingual environment can be acceptably named using either the target language (e.g., me:ju IPA / me:d͡ʒu/) or its translation equivalent/loan word (i.e., table). Poor name agreement with higher concept agreement (that is, multiple names representing a given concept) has been regarded as an index of the richness of languages (Martein, 1995). Most Indian languages are lexically rich as multiple names exist for most nouns (e.g., the “sun” has nearly 10 synonyms in Kannada, Shabdkosh® (2023)) although many are rarely used.

Our data showed the influence of English on the target language (Kannada). Though our participants were multilingual, the names were provided predominantly in Kannada and English. Only five names had local geographical influence (e.g. /bɑːlɖɪ/ (Tulu) – bucket, /bəɪɾɑːs̪/ (Konkani) – towel). However, none was the modal name in contrast to 30% of English names enjoying the modal status (see Table 4 and Fig. 2). The use of words from English can be attributed in part to the historical colonization in India by the English (Schneider, 2018), as well as the increasing dominance of English with globalization (Kalaja & Pitkänen-Huhta, 2020) and advances in communication technologies (Chibaka, 2018; Kelly-Holmes, 2019). When compared with other languages, the use of English as an alternative language has become an essential part of multilingualism (Aronin & Singleton, 2008, 2012; Chengappa, 2001; Kalaja & Pitkänen-Huhta, 2020).

In India, people are generally aware of basic English vocabulary, such as the labels of household articles (e.g., groceries) as they are tagged with their English names. Such awareness is further augmented, for some concepts, by the lack of equivalents in the native language (e.g., guitar). These words become lexicalized to the native language (e.g., “pen” to /pennu/ in Kannada). The previously published picture corpora from India do acknowledge the influence of borrowed words (Singh et al., 2020; Bangalore et al., 2022), albeit briefly (i.e., focussing only on the modal names) and ignoring the nature of these words (i.e., whether or not there is a target language word). Thus, we believe the current analysis is more robust than its predecessors.

The psycholinguistic variables (frequency, familiarity, and age of acquisition) of the noun corpus also showed an apparent influence of the multilingual context. For instance, in our study, the mean familiarity and subjective frequency values were centred around 3. That means the modal names were only moderately frequent and familiar to the multilingual participants of the current study. This is likely, at least in part, due to the well-attested fact that because multilinguals speak in more than one language, inevitably the frequency of use of a word in any of those languages will be lower than that of the same word in monolinguals (e.g., Gollan et al., 2008). Similarly, the age of acquisition of the names varies across the languages of a multilingual speaker, depending on the context of acquisition of each language and the frequency of its use. Hence, the mean age of acquisition of the items in the noun corpus was relatively high for our participants.

## Methodological notes

The *H*-statistic provides a measure of variability in the name agreement responses. Previously, to calculate the *H*-statistic, researchers included “dominant and non-dominant words” (which are both correct to a concept) and excluded the “don’t know and tip of the tongue” responses (Snodgrass & Vanderwart, 1980). In French norms (Alario & Ferrand, 1999) the concept “accordion” had 100% name agreement, with an *H*-statistic of 0, suggestive of perfect name agreement. In our corpus, “accordion” had 16.67% correct responses, and 80% of responses were incorrect naming using the label “harmonium”. However, as this label refers to a different concept this response could be reasonably excluded and the *H*-statistic would therefore be 0 (perfect name agreement). However, we believe that for name agreement data, it is important to consider all names (i.e., incorrect and correct labels). As noted above, most previous studies were not explicit regarding whether they had excluded incorrect concept names in *H*-statistic calculations, although George and Mathuranath (2007) explicitly note that incorrect responses *were* excluded for *H*-statistic calculation. Critically, we have reported the *H*-statistic both with and without incorrect names for transparency and comparison.

There is also a lack of clarity in the literature regarding whether “non-target” responses should be considered as correct responses. For example, Edmonds & Donovan (2014) consider “receipt” as a correct and acceptable response for a picture of a ticket, considering that it was the result of “picture ambiguity”. A similar example from our data is the picture of a pen that elicited “pencil” as the response. While it is tempting to conclude that the target name for this picture should, therefore, be “pencil”, this can cause difficulties in the use of such stimuli. For example, in a clinical study, if following intervention, a participant changed their response from “pencil” to “pen”, this improvement would not be apparent if “pencil” was also coded as a correct response.

Finally, we alert the reader to some of the limitations of our normative data. The first of these is also true of other databases: Our ratings were obtained from university students in an English-medium environment and it is unclear the extent to which the data can generalise to the broader population of (Kannada) speakers, particularly older adults, as well as to those with less exposure to English.

Secondly, while our ratings showed good reliability across participants for subjective frequency, age of acquisition ratings, familiarity for nouns, and image agreement for verbs, the image agreement ratings for nouns and familiarity ratings for verbs showed poor reliability. We are aware of only two instances where reliability has been reported for norms in the literature (Decuyper et al., 2021; Momenian et al., 2021) and neither provides image agreement ratings (or reliability). While Momenian et al. (2021) do provide familiarity ratings, and the reliability of these ratings is higher than ours (ICC .86–.95 depending on participant language background), they do not provide a breakdown of reliability separately across nouns and verbs.

Critically, however, the poor reliability of ratings for one word type rather than the other in our data was unlikely to be due to any participant-related factors, as the ratings were provided by the same participants for nouns and verbs. Nevertheless, it is possible that as these are multicultural, multilingual participants there is a complex interaction between the language background of the participants and their ratings that results in greater variability between participants for one word class relative to the other.

With regard to the poor reliability of image agreement for nouns relative to verbs, this mirrors the relatively more variable (lower *H*-statistic) naming responses to nouns, suggesting that the variability between participants in the name that they associated with a picture also influences the likelihood that the image does not agree with their mental image. It is also clearly the case that the mental images invoked by the concept of an object can differ from one participant to another. For example, there can be natural (e.g., physical: colour, size, shape, texture) variability in the real objects belonging to a conceptual category itself: the concept of a “pen” can refer to pens of several subtypes (e.g., ball pen, fountain pen, sketch pen) and within each subtype several variants (colour, precise shape, texture) are possible. Additionally, the individual association with the real objects may also influence this rating (e.g., a personally used pen vs. the depicted image). It is possible that the variability across speakers for concepts of action-related verbs is lower, although this hypothesis would need experimental support (see Kurland et al., 2014, for a related idea: pictures where different objects are involved [e.g., trousers, shirt] under a particular category (e.g., clothing) elicit the same target verb (e.g., “wear”).

In terms of lower reliability of verb familiarity ratings relative to nouns, it is possible that the limited range of ratings is the likely source of the poor split-half correlations: verb ratings ranged from 4.2 to 4.8 (on a 1–5 scale), whereas noun ratings ranged from ~1.5 to 5.

Nevertheless, given their low reliability across participants, we are reluctant to recommend the use of our image agreement ratings for nouns and familiarity ratings for verbs. While this is clearly less than optimal, it is hard to know the extent to which this is unusual in ratings given that such reliability measures are rarely reported (but see Decuyper et al., 2021; Momenian et al., 2021). Consequently, we advocate that researchers report these measures routinely in the future to glean a better understanding of the extent to which ratings vary within and across participants. In addition, it is important that, as suggested by Mason et al. (2024), a clear protocol is developed for exclusion of problematic data sets and data points in the development of norms (e.g., Brysbaert et al., 2014; Warriner et al., 2013).

Further research that systematically examines the factors influencing the between-subject variability in the ratings (e.g., influence of age, other languages spoken, language of education) would also be valuable. Finally, investigations using these norms to examine the factors that predict picture naming speed in Kannada speakers will be important, although such analyses would be complex given the multiple acceptable responses for each target in a multilingual context.

## Conclusion

We have presented the novel ICMR-Manipal Colour Picture Corpus, comprising 663 nouns and 96 verbs with their name agreement data as well as the ratings on the key psycholinguistic variables from a multilingual (Indian) context. This corpus may be used for clinical and research purposes. Unlike the normative data from studies on monolingual populations, the data from the current study are indicative of the apparent influence of the multilingual nature of the participants. As the world is becoming increasingly multilingual, we believe that further similar studies in other multilingual contexts are required.

## Figures and Tables

**Fig. 3:**
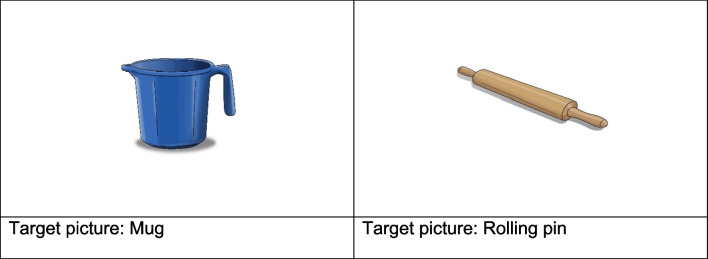
Sample pictures from the noun corpus

**Fig. 4:**
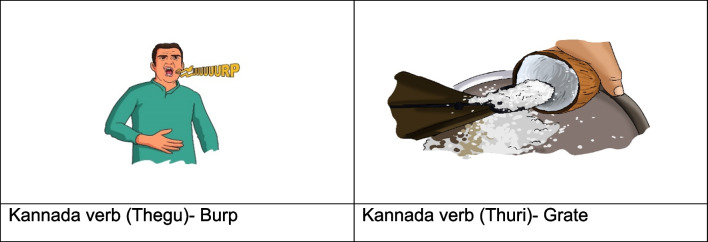
Sample pictures from the verb corpus

## Data Availability

The picture corpus (.jpeg files) and the data set (Excel files) generated from this work are available in the OSF repository: https://osf.io/32m9a/?view_only=6d130363a7cd485890e71b8a74e7a53e.

## Appendix

**Table 5:** Correlation matrix of ratings for the 663 nouns

	Concept agreement (%)	Image agreement	Objective visual complexity	Modal name (%)	H statistic with all response	H statistic for acceptable alternatives	Familiarity	Frequency	Age of acquisition	Syllable count
Concept agreement (%)	—									
Image agreement	0.050	—								
Objective visual complexity	-0.105**	-0.023	—							
Modal name (%)	0.519***	-0.023	-0.124**	—						
H statistic with all response	-0.441***	0.006	0.133***	-0.937***	—					
H statistic for acceptable alternatives	-0.057	0.017	0.089*	-0.758***	0.844***	—				
Familiarity	0.186***	-0.030	-0.016	0.246***	-0.230***	-0.162***	—			
Frequency	0.274***	0.004	-0.145***	0.293***	-0.262***	-0.147***	0.338***	—		
Age of acquisition	-0.307***	0.044	0.056	-0.320***	0.296***	0.178***	-0.268***	-0.327***	—	
Syllable count	-0.036	0.034	0.158***	-0.192***	0.168***	0.149***	-0.106**	-0.282***	0.226***	—

* *p* < .05, ** *p* < .01, *** *p* < .001

**Table 6:** Correlation matrix of ratings for the 96 verbs

	Concept agreement (%)	Image agreement	Objective visual complexity	Modal name (%)	H statistic with all response	H statistic for acceptable alternatives	Familiarity	Frequency	Age of acquisition	Syllable count
Concept agreement (%)	—									
Image agreement	0.180	—								
Objective visual complexity	0.007	-0.012	—							
Modal name (%)	0.692***	0.106	0.075	—						
H statistic with all response	-0.571***	0.009	-0.112	-0.923***—	—					
H statistic for acceptable alternatives	0.103	0.049	-0.068	-0.530***	0.597***	—				
Familiarity	-0.049	0.044	-0.027	-0.138	0.092	0.121	—			
Frequency	0.007	0.016	-0.004	-0.082	0.097	0.148	0.078	—		
Age of acquisition	-0.195	0.309**	0.065	-0.114	0.079	0.056	0.009	0.049	—	
Syllable count	-0.147	-0.185	0.073	-0.030	-0.044	-0.108	-0.317**	-0.166	0.162	—

* *p* < .05, ** *p* < .01, *** *p* < .001

**Table 7:** Syllable count and word classification for the modal names

	Modal name and its word classification			
Syllable count	Cognates	Target language word	Translation Equivalents	Total
1	43	15	37	95
2	92	145	45	282
3	42	93	14	149
4	21	53	10	84
5	1	31	2	34
6	2	9	1	12
7	0	3	1	4
8	0	1	0	1
9	0	1	0	1
10	0	1	0	1
Total	201	352	110	663
	**Value**	**df**	***p***	
χ^2^	83.514	9	< .001	
N	462			

χ^2^ Tests comparing syllable count with target language word and translation equivalents

**Table 8:** Intraclass correlation coefficient (ICC) for reliability of the ratings

	Noun				Verb			
Rating	ICC	Lower 95% CI	Upper 95% CI	Interpretation	ICC	Lower 95% CI	Upper 95% CI	Interpretation
Image agreement	0.143	0.047	0.234	Poor	0.972	0.964	0.98	Excellent
Familiarity	0.637	0.596	0.675	Moderate	0.172	-0.082	0.39	Poor
Frequency	0.985	0.984	0.987	Excellent	0.926	0.904	0.946	Excellent
Age of acquisition	0.982	0.98	0.984	Excellent	0.954	0.94	0.966	Excellent

We calculated the ICC for the ratings received for the modal name. There were 663 nouns and 93 verbs which were rated by 30 participants. Intraclass correlation (Shrout & Fleiss, 1979) was calculated using a two-way mixed effect model with the average type (i.e. mean of k raters). The selection of the type of ICC and its interpretation are based on the guidelines by Koo & Li (2016)

**Table 9:** Split-half correlation comparing the ratings of even (*n*=15) and odd participants (*n*=15)

	Noun		Verb	
Ratings	Spearman's rho	*p*	Spearman's rho	*p*
Image agreement	-0.01	0.805	0.907***	< .001
Familiarity	0.295***	< .001	0.082	0.434
Frequency	0.967***	< .001	0.227*	0.029
Age of acquisition	0.959***	< .001	0.227*	0.029

*p* < .05, ** *p* < .01, *** *p* < .001

Modal names for nouns and verbs were 663 and 93, respectively

## Footnotes

1 The subcategories of human-made (e.g., clothing, vehicles) and natural (e.g., fruits, vegetables) stimuli can be viewed in Supplementary Excel file S2. We adapted Palmer et al. (2017) category list.

2 The verb classification can be viewed in the Supplementary Excel file S3. We used verb classification based on previous studies (Levin, 1993; Vinson & Vigliocco, 2002) as cited in Prarthana (2015).

3 The participants in the name and image agreement task had English as their medium of instruction at the University, and those who rated the words had had either Kannada or English as the medium of instruction at school/college.

4 These are words which are used by many speakers of the language, including people who were not exposed to the English language.

5 d̪uːɾə refers to “tele” and. d̪əɾʃən̪ə indicates “vision”.
